# The Business Case for Bariatric Surgery Revisited: A Non-Randomized Case-Control Study 

**DOI:** 10.1371/journal.pone.0075498

**Published:** 2013-09-19

**Authors:** Eric A. Finkelstein, Benjamin T. Allaire, Denise Globe, John B. Dixon

**Affiliations:** 1 Health Services and Systems Research, Duke-NUS Graduate Medical School, Singapore, Singapore; 2 Global Health Institute, Duke University, Durham, North Carolina, United States of America; 3 RTI International, Durham, North Carolina, United States of America; 4 Global Health Outcomes Strategy and Research, Allergan Inc., Irvine, California, United States of America; 5 Department of General Practice, School of Primary Health Care, Monash University, Melbourne, Australia; 6 Human Neurotransmitters Laboratory, Vascular and Hypertension Unit, The Baker-IDI Heart and Diabetes Institute, Melbourne, Australia; University of Texas Health Science Center at San Antonio, United States of America

## Abstract

**Background and Aim:**

Prior studies reporting that bariatric surgery (including laparoscopic adjustable gastric band (LAGB) and [laparoscopic Roux-en-Y] Gastric Bypass (LRYGB)) is cost-saving relied on a comparison sample of those with a morbid obesity (MO) diagnosis code, a high cost group who may not be reflective of those who opt for the procedures. We re-estimate net costs and time to breakeven using an alternative sample that does not rely on this code.

**Materials and Methods:**

Non-randomized case-control study using medical claims data from a commercial database in the USA. LAGB and LRYGB claimants were propensity score matched to two control samples: one restricted to those with a MO diagnosis code and one without this restriction.

**Results:**

When using the MO sample, costs for LAGB and LRYGB are recovered in 1.5 (Confidence Interval [CI]: 1.45 to 1.55) and 2.25 years (CI: 2.07 to 2.43), and 5 year savings are $78,980 (CI: 62,320 to 100,550) for LAGB and $61,420 (CI: 44,710 to 82,870) for LRYGB. Without the MO requirement, time to breakeven for LAGB increases to 5.25 (CI: 4.25 to 10+) years with a 5 year net cost of $690 (CI: 6,800 to 8.400). For LRYGB, time to breakeven exceeds 10 years and 5 year net costs are $18,940 (CI: 10,390 to 26,740).

**Conclusions:**

The net costs and time to breakeven resulting from bariatric surgery are likely less favorable than has been reported in prior studies, and especially for LRYGB, with a time to breakeven of more than twice the 5.25 year estimate for LAGB.

## Introduction

Severely obese individuals are at greater risk of diabetes and other chronic conditions and have annual expenditures that far exceed expenditures for those with a normal BMI [1,2]. Bariatric surgery, including gastric banding and gastric bypass surgery, is the most effective treatment for severe obesity. Both procedures, which are typically performed laparoscopically, have been shown to generate significant weight loss, improvements in comorbidities, most notably diabetes [3,4], and have been shown to be cost-effective [5,6]. Although average costs in the USA for the procedures are roughly $20,000 for the laparoscopic adjustable gastric band (LAGB) and $24,000 for the [Laparoscopic Roux-en-Y] Gastric Bypass (LRYGB), it is possible that the reduction in comorbidities may lead to a net cost savings [7,8].

Although no randomized studies have been conducted to test this hypothesis, several case control studies have explored the net cost implications [7,9-11]. Three of these studies found that costs were recovered in 4 years or less. Contrarily, Maciejewski et al [11], focusing on mostly older male patients who received gastric bypass surgery at Veterans Administration (VA) medical centers, found no evidence of reduced health expenditures post surgery. A primary difference with their study is that it is the only one that did not rely on the use of an MO diagnosis code to identify controls. The MO code is largely given to those with BMIs well above 40 who are likely to present with medical conditions that can often lead to rapid escalation of costs. As such use of this code may not be appropriate to identify an appropriate control group of bariatric surgery patients.

The aim of this study is to generate net costs and time to breakeven estimates for private sector LAGB and LRYGB patients using a comparison sample not restricted to the MO diagnosis code but propensity score matched to the surgery samples on other observable demographic and comorbidity variables. We hypothesize that results based on this sample will yield greater net costs of bariatric surgery and a longer time to breakeven.

## Materials and Methods

### Data

The analysis is based on claims data from the MarketScan® Commercial Claims and Encounters database between January 1, 2003, and September 31, 2009.

The database includes de-identified, person-specific inpatient, outpatient, and retail pharmaceutical claims from approximately 100 large payers representing millions of covered lives. The claims include visit-level information, including dates of service, diagnosis and procedure codes, and payments. For the surgically treated patients it includes the costs of surgical follow-up and complications. A linked patient-level file provides additional demographic data, including age and gender, type of health plan, and periods of eligibility [12]. The RTI International IRB deemed that the study, involving secondary data analyses on bariatric surgery using MarketScan® data, was exempt from review based on the criteria that the research involved existing data where information was recorded in a manner where subjects could not be identified either directly or through identifiers linked to the subjects.

### Sample Selection

The matching strategy began with the LAGB sample. The approach for selecting this sample has been discussed previously [10]. In brief, all persons aged 18 to 64 with evidence of a banding placement code (Healthcare Common Procedure Code [HCPCS] S2082 and Current Procedural Terminology [CPT] code 43770) and no evidence of stomach or intestinal cancers (Internal Classification of Disease 9th Edition [ICD-9] codes 150–159 and 230) were included. Persons who did not have one of these two codes but had evidence of either a banding adjustment or removal (HCPCS code S2083; CPT codes 43771, 43772, 43773, and 43774; or ICD codes 44.96, 44.97, or 44.98) were also included if an initial LAGB procedure date could be identified using a wider set of bariatric procedure codes commonly used before 2004. If none of these codes were identified, or if there were multiple bariatric procedure codes on different dates, then the individual was not included in the analysis.

The LRYGB sample was identified using the following codes: 43644 and 43645 (CPT), S2085 (HCPCS), or 44.38 (ICD-9). For both surgery samples, the date on which the initial procedure occurred is indexed as the surgery date. The MO comparison sample was identified using ICD-9 code V85.4 for “BMI 40 and over, adult.” The random sample of 120,000 individuals was provided directly from Medstat. For all samples, the subset with diabetes was defined as individuals with at least two outpatient claims or one inpatient claim with a diagnosis of diabetes (ICD 250).

### Analysis

Propensity score matching was used to ensure that the four groups were as similar as possible. LRYGB patients were matched to LAGB patients based on patient and health plan characteristics, and on diagnoses and costs in the year prior to the quarter before the bariatric procedure. This 1-year window was used to ensure that the claims occurring in the buildup to the procedure did not influence the match. Operationally, the LAGB and LRYGB samples were combined and a probit model was used to predict the probability (propensity) of LAGB (as opposed to LRYGB) as a function of observable characteristics. The variables used in the match included age, gender, type of health plan (Health Maintenance Organization [HMO], Preferred Provider Organization [PPO], or comprehensive), year of surgery, presence of obesity related or highly prevalent comorbidities (including diabetes, asthma, arthritis, hypertension, sleep apnea, dyslipidemia, migraines, and chronic obstructive pulmonary disease), and inpatient and total costs in each quarter of the match period.

Matching the MO and random samples to the LAGB sample occurred similarly; however, before the match could occur, the match period needed to be defined for the comparison samples as they did not have a surgery date to reference. This was resolved by assigning each individual in the control samples a pseudo-surgery start date by randomly drawing from the distribution of surgery dates for the LAGB sample. To further improve the match and reduce the influence of outliers, cases with an inpatient admission in the quarter prior to the (pseudo) surgery date were removed out of concerns that comparison sample cases who had an inpatient admission in this quarter might be unlikely to have a bariatric procedure in the following quarter. The most expensive 2.5% of cases from each sample were also removed to minimize the influence of high cost and potentially unrelated medical events on the results.

Nearest neighbor and one-to-one matching without replacement was employed based on each individual’s predicted value (propensity) for LAGB from the probit model. This approach ensured that all samples matched the comorbidity profile of the LAGB sample. An identical approach was conducted for the diabetes subsample. However, it was necessary to match to the LAGB sample with replacement to create a suitable match.

Using the four matched samples, an analysis dataset was created that included quarterly payments of total, inpatient (both facility and physician), non-inpatient (including payments for hospital outpatient, physician’s office visits, and emergency department), and prescription drug claims. Each quarter represented the time relative to (pseudo) band placement. All payments were inflated to two thousand and eleven dollars using the medical care component of the Consumer Price Index. For each individual, data were dropped from all quarters where a person was not fully enrolled. Differences in the (pseudo) surgery date and duration of enrollment resulted in different observation lengths for each individual.

A maximum likelihood Tobit model was used to compare quarterly payments for the LAGB, LRYGB, and control samples. The Tobit model is appropriate because medical expenditures are non-negative [13]. The model specification included quarterly dummy variables and three dummy variables representing LRYGB, MO, and random sample claimants, with LAGB as the omitted reference group. To test for the costs of LAGB relative to LRYGB or the controls in each quarter, the model also included interactions between each quarterly dummy variable and the LRYGB, MO, and matched random sample indicators. Because the overall and diabetes samples become small after quarters 16 and 12, respectively, data were pooled beyond these quarters and a single indicator was used to represent average quarterly costs for these quarters and beyond.

The cost implications of LAGB can be quantified by using the regression results to compare quarterly payment differences between the LAGB, LRYGB, and comparison samples beginning the quarter before (pseudo) surgery. The present value of these payments in subsequent quarters, from the time of initial placement, represents the net costs and was calculated using an annual discount rate of 3%. Five-year net costs and time to breakeven are presented based on 1,000 bootstrapped replications. All analyses were conducted using Stata 11.1 [14].

## Results

Table 1 presents summary statistics. 9,651 LAGB patients met the inclusion criteria. These patients are predominantly female and average 44 years old at the time of surgery. Roughly 25% have diabetes, and the prevalence of comorbidities ranged from 8.4% for asthma to 45% for arthritis. Payments for the LAGB sample in the year before the quarter before surgery averaged $9,971.

**Table 1:** Panel A. Samples Prior to Matching and Trimming.

	**Full Sample**				**Diabetes Subsample**			
**Population**	**LAGB**	**LRYGB**	**Random Sample**	**Morbid Obesity Sample**	**LAGB**	**LRYGB**	**Random Sample**	**Morbid Obesity Sample**
Observations	9,651	21,533	120,175	10,907	2,455	6,646	6,850	2,461
Age (Years)	44.2	44.5*	44.0+	47.1*+	49	48.8	50.5*+	52.2*+
Female (%)	79.1	79.4	68.3*+	65.7*+	71.2	72.3	62.5*+	60.0*+
Health plan: PPO (%)	56.3	49.1*	52.6*+	51.5*+	57.8	51.7*	55.1*+	53.5*
Health plan: HMO (%)	14.6	24.5*	28.4*+	29.5*+	14.1	22.4*	25.5*+	25.9*+
Health plan: comprehensive (%)	6.6	7.5*	3.5*+	4.3*+	6.5	7.4	4.9*+	5.8+
Diabetes (%)	25.4	30.9*	5.7*+	22.6*+	1	1	1	1
Asthma (%)	8.4	9.7*	2.7*+	7.8+	9.1	9.6	9	9
Arthritis (%)	44.8	45.6	22.7*+	42.7*+	49.3	49.8	50.2	52.9*+
Hypertension (%)	44.2	44.5	15.4*+	28.8*+	58.5	56.5	56.9	56.6
Sleep apnea (%)	20	24.4*	2.6*+	11.5*+	24.9	29.7*	16.4*+	18.1*+
Migraines (%)	9.5	9.2	4.3*+	1.4*+	7.5	7	7.5	7.8
Dyslipidemia (%)	31.6	31	17.8*+	7.9*+	45.7	44.2	47.2+	49.5*+
COPD (%)	4.9	5.9*	1.8*+	5.8*	6.4	7.5	7.7*	8.7*
Inpatient payments (Q -2)	299.6	282.2	181.2*+	1,321.9*+	459.7	428.8	1,025.1*+	2,009.9*+
Inpatient payments (Q -3)	353.8	345.8	268.3	773.0*+	468.5	485.9	1,202.9*+	1,273.2*+
Inpatient payments (Q -4)	342.1	375.3	236.0*+	517.9*+	459.9	529	1,137.4*+	856.9*+
Inpatient payments (Q -5)	451.8	453.3	204.3*+	573.6+	704.1	597.3	1,003.9*+	797.3
Total payments, 1 year before procedure	9,971.30	10,554.3*	4,831.4*+	11,497.1*+	14,612.60	14,628.30	16,257.0*+	17,887.5*+

^*^ Different from the LAGB group at the 95% level.

^+^ Different from the LRYGB group at the 95% level.

Notes: COPD = chronic obstructive pulmonary disease; HMO = health maintenance organization; LAGB = laparoscopic adjustable gastric band; LRYGB = Laparoscopic Roux-en-Y Gastric Bypass; PPO = preferred provider organization; Q = quarter

Relative to LAGB patients, LRYGB patients are slightly older and have a different health plan mix and distribution across years. They also have higher rates of diabetes (30.9% vs. 25.4%), asthma (9.7% vs. 8.4%), and sleep apnea (24.4% vs. 20.0%) and greater annual payments in the year prior to the surgery quarter ($10,554 vs. $9,971).

The MO sample is roughly 3 years older than the LAGB sample and has a smaller percentage of females (65.7% vs. 79.1%). The health plan mix and distribution across years also differs. Moreover, although the prevalence of the included comorbidities is statistically lower than in the surgery samples, the annual costs are more than $1,500 greater for the MO sample. This suggests that other differences are making this sample more expensive.

The random sample differed markedly from all three samples. This sample had a lower proportion of females and a different health plan mix. It was also far healthier and had costs that were less than half of the costs for the other samples. Within each sample, the subset with diabetes tended to be older, sicker, more expensive, and more likely to be male. Relative differences across the four samples were similar.

Table 2 reveals that after propensity score matching, for the full samples there were no statistically significant differences between the LRYGB, random, and LAGB samples. Although the propensity scoring approach was unable to increase the comorbidity profile of the MO sample to fully match the surgery samples, post matching there were no statistically significant differences in costs in the reference period, suggesting the match may still be appropriate. To the extent that the MO sample may be slightly healthier, this would bias the estimates toward a longer breakeven period for the surgeries. Among the subset with diabetes, post matching there were no statistically significant differences in costs or comorbidities across samples.

**Table 2:** Panel A. Samples After Matching and Trimming.

	**Full Sample**				**Diabetes Subsample**			
**Population**	**LAGB**	**LRYGB**	**Random Sample**	**Morbid Obesity Sample**	**LAGB**	**LRYGB**	**Random Sample**	**Morbid Obesity Sample**
Observations	9,631	9,631	9,631	9,639	2,447	2,447	2,447	2,455
Age (Years)	44.2	44.1	44.2	46.5*+	49	48.9	48.9	48.4*
Female (%)	79.2	78.9	79.3	70.1*+	71.3	71	72.8	70.9
Health plan: PPO (%)	56.4	55.6	55.9	54.6*	57.9	56.9	57.5	59.7
Health plan: HMO (%)	14.7	14.3	14.2	24.5*+	14.1	13.5	13.9	12.8
Health plan: comprehensive (%)	6.6	7.6*	7	4.7*+	6.5	7	6	7.3
Diabetes (%)	25.4	25.6	25.7	23.5*+	1	1	1	1
Asthma (%)	8.5	8.6	8.7	7.8+	9.1	9.8	9.6	8.9
Arthritis (%)	44.8	44.9	45.3	43.5	49.2	50.5	49.4	49.4
Hypertension (%)	44.2	44	44.6	38.9*+	58.4	57.1	57.2	57.6
Sleep apnea (%)	20	20.4	20	12.6*+	24.7	24.3	25.2	24.3
Migraines (%)	9.5	9.6	9.6	8.5*+	7.4	7.2	7.6	8
Dyslipidemia (%)	31.6	31.1	31.4	30.0*	45.7	46.2	43.6	46.3
COPD (%)	4.9	4.9	5.4	5.7*+	6.3	5.6	6.5	5.5
Inpatient payments (Q -2)	269.90	278.00	279.2	536.1*+	422.10	516.30	529.3	522.3
Inpatient payments (Q -3)	349.50	375.70	404.2	569.8*+	466.20	389.50	494.6	411.8
Inpatient payments (Q -4)	342.50	337.60	345.6	395	461.50	385.90	403.2	504
Inpatient payments (Q -5)	425.80	499.50	384.6+	531.3	658.60	493.00	686	425.4
Total payments, 1 year before procedure	9,765.80	10,101.80	9912.8	10167.1	13,909.30	14,107.70	14274.3	13706.2

* Different from the LAGB group at the 95% level.

^+^ Different from the LRYGB group at the 95% level.

Notes: Matching was conducted “without replacement,” meaning that each comparison sample patient was represented once in the analysis. COPD = chronic obstructive pulmonary disease; HMO = health maintenance organization; LAGB = laparoscopic adjustable gastric band; LRYGB = Laparoscopic Roux-en-Y Gastric Bypass; PPO = preferred provider organization; Q = quarter

**Figure 1:**
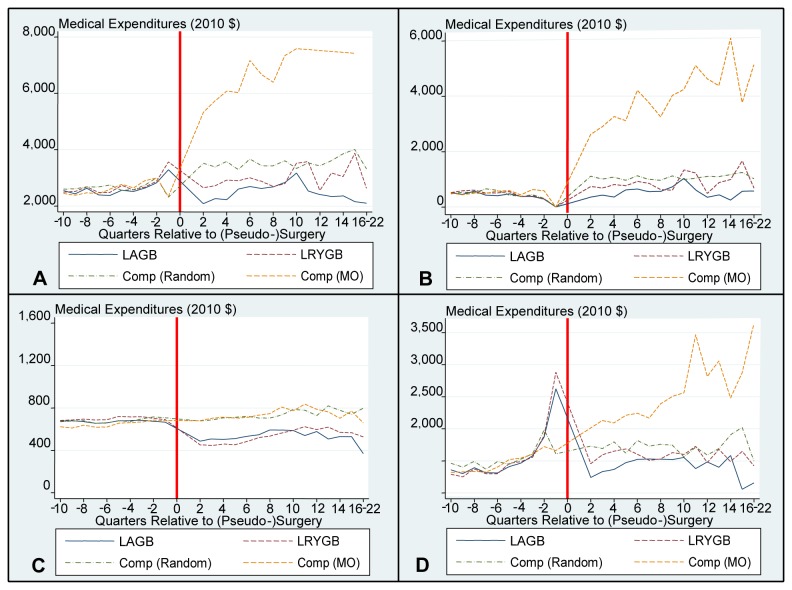
Full Sample – Mean Medical Expenditures By Quarter. Panel A illustrates Mean Total Payments after matching in the Full Sample. Panel B illustrates Mean Inpatients Payments after matching in the Full Sample. Panel C illustrates Mean Outpatient Payments after matching in the Full Sample. Panel D illustrates Mean Pharmaceutical Payments after matching in the Full Sample. For scaling purposes, Q1 costs have not been included in the graph. The surgical date has been replaced with a red line. Comp is short for comparison sample; MO is short for morbid obesity.

**Figure 2:**
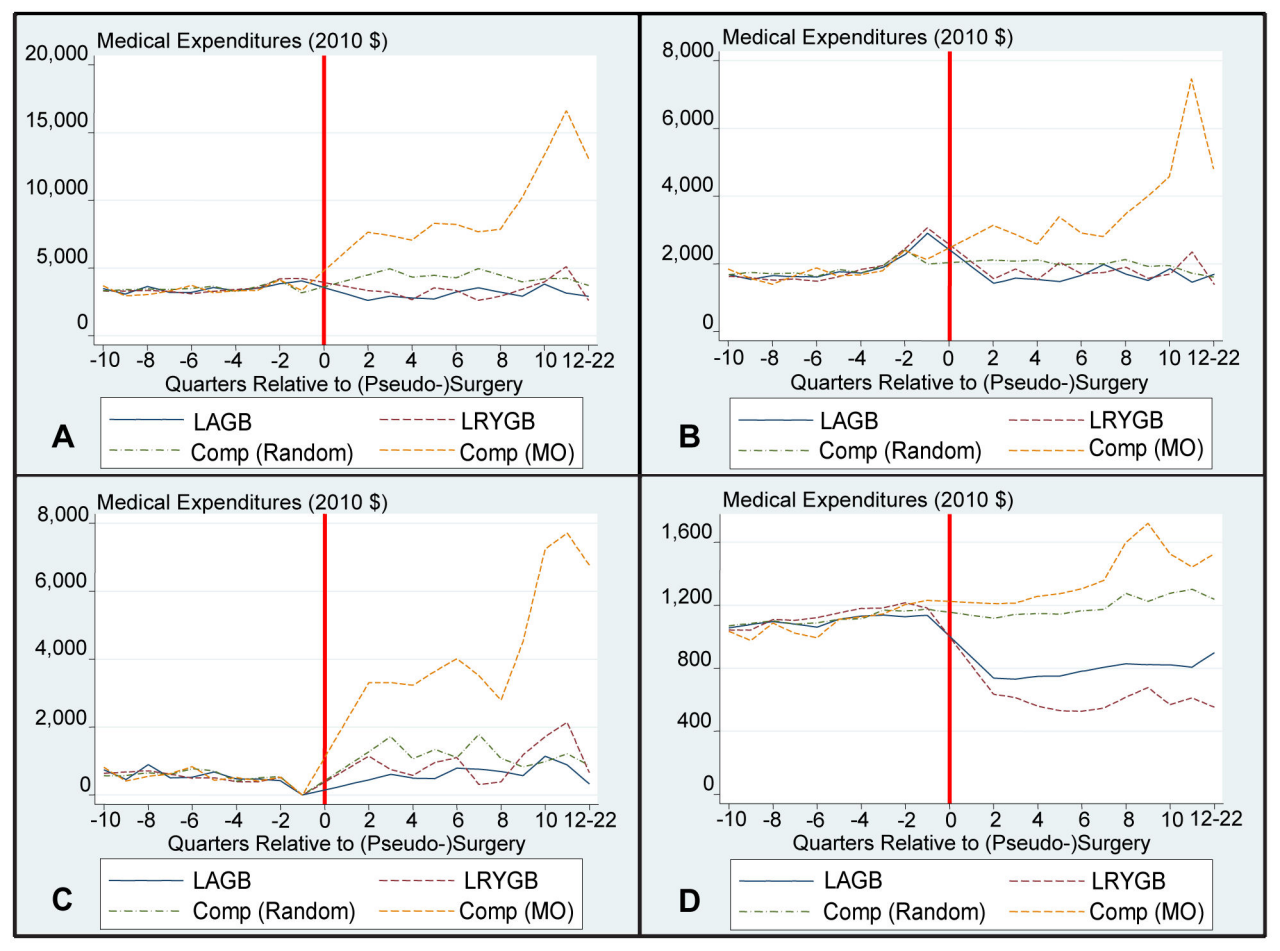
Diabetes Subsample – Mean Medical Expenditures By Quarter. Panel A illustrates Mean Total Payments after matching in the Diabetes Sample. Panel B illustrates Mean Inpatients Payments after matching in the Diabetes Sample. Panel C illustrates Mean Outpatient Payments after matching in the Diabetes Sample. Panel D illustrates Mean Pharmaceutical Payments after matching in the Diabetes Sample. For scaling purposes, Q1 costs have not been included in the graph. The surgical date has been replaced with a red line. Comp is short for comparison sample; MO is short for morbid obesity.

Figures 1 and 2 provide graphical representations of the cost trends pre- and post- (pseudo) surgery for total, inpatient, outpatient, and pharmaceutical costs. The surgery quarter, Q1, is not included on these graphs as including this quarter would reduce the scale to the extent that trends would not be observable. Costs for the LAGB and LRYGB samples in this quarter were $21,980 and $29,900 for the full sample and $22,480 and $31,150 for the diabetes subsample, respectively. These figures reveal a slight increase in costs for the surgery samples in the run-up to surgery.

Additionally, there is a reduction in costs from trend in the quarter after surgery. The largest relative effects occur for pharmaceutical payments, where costs decrease in quarter 2 for the full samples and diabetes subsamples and remain lower than pre-surgery costs throughout the analysis period. This decrease is largely driven by a reduction in costs for diabetes medications (results available upon request).

There is no reduction from trend for the matched random sample, whose costs increase in a linear fashion throughout the analysis period. Costs for the MO sample immediately escalate post pseudo surgery, largely driven by a sharp increase in inpatient costs, thus revealing significant underlying differences between this and the matched random sample. This increase in MO costs is driven by higher rates of admissions. Roughly one-third of the MO sample had an admission post pseudo-surgery, whereas this figure is 10% for the remaining samples (results available upon request).

Table 3 presents the time to breakeven and 5-year net costs. When compared to the MO sample, costs for LAGB and LRYGB appear to be fully recovered in 1.5 (Confidence Interval [CI]: 1.45 to 1.55) and 2.25 years (CI: 2.07 to 2.43), respectively. As a result, these procedures appear to generate significant savings at 5 years: $78,980 (CI: $62,320 to $100,550) for LAGB and $61,420 (CI: $44,710 to $82,870) for LRYGB. Some of the difference in savings between the two procedures results from the higher estimated surgical costs for LRYGB ($16,680 vs. $22,140). Results appear even more compelling for the diabetes subsample, with costs fully recovered in 1.25 (CI: 1.02 to 1.48) years for LAGB and 1.75 (CI: 1.49 to 2.01) years for LRYGB and even larger estimated savings at 5 years; $127,590 (CI: $94,840 to $167,590) for LAGB and $103,340 (CI: $65,550 to $146,760) for LRYGB.

**Table 3:** Time to Breakeven and Net Costs for Full and Diabetes Samples.

	**Time to Breakeven (Years**)		**5-year Net Costs (United States Dollars**)	
**Morbid Obese Sample**				
**Sample**	**LAGB**	**LRYGB**	**LAGB**	**LRYGB**
Full sample	1.5 (1.45 1.55)	2.25 (2.07 2.43)	−78,980 (−100,550-62,320)	−61,420 (−82,870-44,710)
Diabetes subsample	1.25 (1.02 1.48)	1.75 (1.49 2.01)	−127,590 (−167,590-94,840)	−103,340 (−146,760-65,550)
**Random Sample**				
**Sample**	**LAGB**	**LRYGB**	**LAGB**	**LRYGB**
Full sample	5.25 (4.25 10+)	10+	690 (−6,800 8,400)	18,940 (10,390 26,740)
Diabetes subsample	4.25 (3 10+)	10+	−3,060 (−13,230 7,930)	21,610 (3,330 42,570)

Note: LAGB = laparoscopic adjustable gastric band; LRYGB = Laparoscopic Roux-en-Y Bypass

Figures 1 and 2 reveal that when comparisons are made to the matched random sample, the estimated time to recover the costs of a LAGB procedure increases to 5.25(CI: 4.25 to 10+) years for the full sample. Five-year net costs (not savings) are $690 (CI: $-8,400 to $6,800). For LRYGB net costs at 5 years are $18,940 (CI: $10,390 to $26,740). Based on projections, it would take more than 10 years to recover the costs of the LRYGB procedure.

Focusing on the diabetes subsample, when comparing to the matched random sample the estimated time to recover the costs of a LAGB procedure is 4.25 (CI: 3 to 10+) years and five-year net costs are now negative, revealing a savings of $3,060 (CI: $-7,930 to $13,230). For LRYGB, the net costs remain positive (i.e., no savings) at 5 years; $21,610 (CI: $3,330 to $42,570) and, based on projections, it would again take more than 10 years to recover the costs of the procedure.

## Discussion

The return on investment for bariatric surgery, be it banding or bypass, depends on 3 factors: 1) the cost of the surgical procedures, 2) the subsequent cost profile among those who undergo the procedure, and 3) what their costs would have been in the absence of the surgical intervention. Concerning points 1 and 2 there is near universal agreement as this information is readily available from claims data. The challenge is predicting what costs would have been in the absence of surgery. Without the benefit of a randomized trial, researchers have had to construct convenience samples that they assume mirror the experience of surgery patients had they not undergone the procedure. Because BMI data is not typically available in claims data, researchers have used those with an MO code as a proxy for the surgery eligible population. This group is indeed eligible for surgery, however, they may be a very high cost/high risk subset of the surgery eligible population such that their subsequent health care experience is not truly reflective of what the surgery population would have experienced in the absence of the procedure. This is likely to be the case as those with an MO code are a small subset of the BMI 40+ population who typically were assigned this code as the result of an inpatient visit that had obesity related complications.

The matched random sample, by matching on comorbidities, may be more reflective of the health and cost trajectory of the surgery sample in the absence of the procedure. As additional evidence to this point, Table 4 compares the age and prevalence of three primary risk factors for obesity from our matched random sample to two surgery eligible samples, those with a BMI of 35 to less than 40 and 40+ from the nationally representative Medical Expenditure Panel Survey. Although we are unable to ascertain the exact BMI of our matched random sample from the claims data, this table shows that the matched random sample is slightly older and has a greater prevalence rate of each of the three primary comorbidities than even the BMI 40+ population. As such, this group almost surely has an average BMI of at least 35 and because there is no MO code indicating they are at especially high risk of deteriorating health, may be a better representation of costs for surgery eligible individuals in the absence of the procedure. As such, the net benefits for bariatric procedures may be best seen by a comparison to this sample.

**Table 4:** Comparison of Comorbidities from the Matched Random Sample and from the Medical Expenditure Panel Survey.

**Population**	**MEPS data (BMI≥18.5 &<25**)	**MEPS data (BMI≥25 &<30**)	**MEPS data (BMI≥30 &<35**)	**MEPS data (BMI≥35 &<40**)	**MEPS data (BMI≥40**)	**Random Sample (After Matching**)
Observations	24,528	24,728	12,627	4,973	3,169	9,631
Age (Years)	37.4	41.4	42.6	42.7	42.5	44.2
Diabetes (%)	2.5	5.1	10.3	16.6	21.0	25.7
Hypertension (%)	8.6	17.4	27.5	34.3	42.9	44.6
Dyslipidemia (%)	7.3	14.8	19.8	21.0	20.2	31.4

Consistent with this hypothesis, use of the matched random sample leads to more conservative estimates of the net costs of the procedures. For LRYGB, the time to breakeven increases from less than 2.25 years for the MO sample (1.75 years for the diabetes subsample) to well beyond 10 years for the matched random sample for both the full and diabetes subsamples. Because of the small sample size and out of sample forecasting required, an actual time to breakeven was not reported. Regardless of the time to breakeven, it is worth pointing out that the expectation for any surgical intervention to show a return on investment is unusual and few effective interventions reach this threshold. LAGB, however, may be one of the exceptions.

For LAGB, net costs and time to breakeven were also less favorable using the matched random sample. However, even with this sample, the time to breakeven was just over 5 years for the full sample and 4.25 years for the diabetes subsample. Without a randomized trial we cannot definitively say which, if any, of these results is closest to reality, however, these findings, combined with the results of Maciejewski et al [11], suggest that results based on the random sample are likely more accurate.

This analysis is subject to several limitations. First, in addition to how the comparison sample is constructed, the net costs and time to breakeven were also influenced by the choice of the matching variables, the determination of the pseudo surgery date and matching period for the controls, the elimination of extremely high cost cases, and whether the match occurred with or without replacement. However, for each strategy employed, restricting the match to those with an MO code reduced the net costs and improved the time to breakeven (results available upon request). Excluding the highest 2.5% of cases from each group increased the expected time to breakeven. Although the groups were matched on a number of attributes, it was not possible to match on BMI because this information was not available in the data. However, because it is not limited to VA data, it is more generalizable than the study by Maciejewski et al [11].

In conclusion, these results reveal that the net costs and time to breakeven resulting from bariatric surgery are less favorable than has been reported in prior studies. Yet, even with a more conservative and likely more accurate comparison sample, the business case for LAGB appears favorable. Regardless, the decision of which procedure is right for a given individual depends on many factors, although cost is likely to be a significant consideration. 
